# Temperatures Outside the Optimal Range for *Helicobacter pylori* Increase Its Harboring within *Candida* Yeast Cells

**DOI:** 10.3390/biology10090915

**Published:** 2021-09-15

**Authors:** Kimberly Sánchez-Alonzo, Luciano Arellano-Arriagada, Susana Castro-Seriche, Cristian Parra-Sepúlveda, Humberto Bernasconi, Héctor Benavidez-Hernández, Víctor L. Campos, Katia Sáez, Carlos T. Smith, Apolinaria García-Cancino

**Affiliations:** 1 Laboratory of Bacterial Pathogenicity, Department of Microbiology, Faculty of Biological Sciences, Universidad de Concepcion, Concepción 4070386, Chile; kimsanchez@udec.cl (K.S.-A.); lucarellano@udec.cl (L.A.-A.); sucastro@udec.cl (S.C.-S.); cparras@udec.cl (C.P.-S.); hebenavides@udec.cl (H.B.-H.); csmith@udec.cl (C.T.S.); 2 Laboratorio Pasteur, Research and Development Area, Concepción 4030000, Chile; hbernasconi@lpasteur.cl; 3 Laboratory of Environmental Microbiology, Department of Microbiology, Faculty of Biological Sciences, Universidad de Concepción, Concepción 4070386, Chile; vcampos@udec.cl; 4 Department of Statistics, Faculty of Physical and Mathematical Sciences, Universidad de Concepción, Concepción 4070386, Chile; ksaez@udec.cl

**Keywords:** *Helicobacter pylori*, intracellular, *Candida*, stress, temperature

## Abstract

**Simple Summary:**

*Helicobacter pylori* is associated with the development of diverse gastric pathologies. This bacterium has been shown to invade yeast to protect itself from environmental factors such as changes in pH, the presence of antibiotics or variations in nutrients that affect their viability. However, intra-yeast *H. pylori* has been reported from other sources, including food, or when the storage temperature is outside the optimal growth range for *H. pylori*, which is 30–37 °C. It is necessary to continue investigating the environmental factors that participate in the entry of the bacteria into yeast. In this work, it was evaluated whether temperature changes promote the entry of *H. pylori* into *Candida* and whether this endosymbiosis favors bacterial viability. It was observed that *H. pylori* significantly increased its invasiveness to yeast when these two microorganisms were co-cultured under 40 °C. The results support that *H. pylori* invades yeasts to protect itself from stressful environments, favoring its viability in these environments. In addition, it can be suggested that this microorganism would use yeast as a transmission vehicle, thereby contributing to its dissemination in the population. However, the latter still needs to be confirmed.

**Abstract:**

*Helicobacter pylori* is capable of entering into yeast, but the factors driving this endosymbiosis remain unknown. This work aimed to determine if temperatures outside the optimal range for *H. pylori* increase its harboring within *Candida*. *H. pylori* strains were co-cultured with *Candida* strains in Brucella broth supplemented with 5% fetal bovine serum and incubated at 4, 25, 37 or 40 °C. After co-culturing, yeasts containing bacteria-like bodies (Y-BLBs) were observed by optical microscopy, and the bacterium were identified as *H. pylori* by FISH. The *H. pylori 16S rRNA* gene was amplified from the total DNA of Y-BLBs. The viability of intra-yeast *H. pylori* cells was confirmed using a viability assay. All *H. pylori* strains were capable of entering into all *Candida* strains assayed. The higher percentages of Y-BLBs are obtained at 40 °C with any of the *Candida* strains. *H pylori* also increased its harboring within yeast in co-cultures incubated at 25 °C when compared to those incubated at 37 °C. In conclusion, although *H. pylori* grew significantly at 40 °C, this temperature increased its harboring within *Candida*. The endosymbiosis between both microorganisms is strain-dependent and permits bacterial cells to remain viable under the stressing environmental conditions assayed.

## 1. Introduction

*Helicobacter pylori* is a Gram-negative, microaerophilic bacterium whose optimum growth temperature is in the range of 35–37 °C [1]. This pathogen colonizes the human stomach [2]. Moreover, it has been detected, by molecular techniques, in diverse water sources and food (including milk and fruits). Nevertheless, reports of the isolation of this pathogen by culture techniques from the same sources previously mentioned are scarce [3,4,5,6]. For example, it has been reported that this bacterium can remain viable for 9–11 days in vitro *H. pylori*-contaminated milk [4], possibly because it acquires a viable but not culturable (VBNC) condition when it is subjected to stressing factors, such as changes of temperature, pH or exposure to oxygen, making it difficult to isolate it in culture media [7,8]. Several environmental factors affect microbial growth rate, the temperature being among the key ones. Therefore, temperature variations can be considered as one of the main stressing factors to which microorganisms are subjected. Temperature affects, among others, the fluidity and function of the cell membrane, gene expression, enzymatic activity and transport of nutrients. Thus, adaptation to temperature is crucial for microorganisms to remain viable [9,10,11], with *H. pylori*, the bacterium subject of the present work, not being an exception.

*H. pylori* is a microorganism showing a high worldwide prevalence, ranging from 85 to 95% in developing countries and 30–50% in developed countries [12,13,14]. These are alarming percentages considering all the gastric and extra-gastric pathologies associated with the infection with this pathogen [15,16] and its increased antibiotic resistance [17,18]. Moreover, in 2017, *H. pylori* were included by the WHO in the list of priority pathogens resistant to antibiotics, and it was classified as priority 2: high [19]. The last Maastricht consensus established that in case of the presence of *H. pylori* in a patient, even in asymptomatic cases, the microorganism must be eradicated in order to reduce the incidence of the pathologies associated with this bacterium [20].

Although *H. pylori* has been studied for more than 35 years [21], still, several questions remain regarding its dissemination, protection and evasion to treatments against its infection mechanisms [22,23]. This is related to several survival mechanisms, among them its ecological niche, the gastric environment, which considerably reduces the action of antibiotics that scarcely diffuse in the mucosa covering this anatomical site and the inactivation of these compounds due to the stomach pH [24]. Additionally, this microorganism is capable of developing a coccoid stage, a resistance strategy, or forming biofilms; both conditions allow its survival in stressing environments, such as the presence of antibiotics, deficiency of nutrients or acidic pH [8,24,25,26,27]. In addition, *H. pylori* has the ability to harbor within eukaryotic cells when subjected to stressing conditions [27,28,29,30,31], a mechanism making harder its eradication and also favoring its dissemination. In this sense, *H. pylori* may remain viable within free-living amoebas isolated from water sources [32,33,34]. Another microorganism postulated as a possible shelter and transmission vehicle for *H. pylori* are yeasts, which are present both in humans and food [6,35,36,37,38]. Yeasts are capable of adapting to different environmental conditions, such as deficiency of nutrients or pH and temperature fluctuations [39]. With respect to yeasts belonging to the genus *Candida*, considered as opportunistic pathogens, it is known that they successfully colonize the human body and that they are part of the normal mouth, skin and genitals microbiota of some healthy individuals [40,41,42]. They can grow under various environmental conditions, including a wide range of temperatures (20–42 °C) [43], which might protect intra-yeast *H. pylori* cells from changes in this environmental factor.

The literature has provided evidence of a close relationship between *H. pylori* and yeast cells belonging to the genus *Candida*. Ansorg and coworkers provided, in 1998, one of the earliest reports on this relationship [44]. The presence of these two microorganisms in gastric ulcers triggers a severe manifestation of this pathology [45,46], suggesting that the interaction between these two microorganisms may be more relevant than that so far recognized. Nevertheless, despite the importance of the relationship between intra-yeast *H. pylori* and *Candida*, there is still a significant lack of knowledge on this relationship which is required to be investigated. Considering the particular interest of our research group to investigate the environmental factors which may promote the internalization of this pathogenic bacterium within yeasts [47,48,49], the aim of the present work was to determine if temperatures outside the optimal growth rate of *H. pylori* promote its internalization into yeasts of the genus *Candida* and to determine if the temperature-triggered bacterium yeast endosymbiotic relationship is strain-dependent.

## 2. Materials and Methods

### 2.1. Strains

In this work, *H. pylori* and *Candida* strains were used. Regarding *H. pylori*, the strains used were SS-1, G-27, ATCC 700824 (often referred to as J99) and H707. The first three are *H. pylori* reference strains, and the latter was obtained from a gastric biopsy. With respect to *Candida*, two reference strains C. *glabrata* ATCC 90030 and *C. albicans* ATCC 90028, and two clinical strains, obtained from a vaginal flow (*C. albicans* VT-3) and from the oral cavity (*C. glabrata* LEO-37), were used. All these strains are maintained at the Laboratory of Bacterial Pathogenicity, Department of Microbiology, Faculty of Biological Sciences, University of Concepcion, Chile.

### 2.2. Strains Culture

*H. pylori* was cultured in Columbia agar (CA) (OXOID, Basingstoke, UK) supplemented with 5% fetal bovine serum (FBS) (Biological Industries, Cromwell, CT, USA) and incubated under microaerobic conditions (Thermo Scientific, Waltham, MA, USA) at 37 °C for 48–72 h. *Candida* strains were cultured in Sabouraud agar (SA) (Merck, Darmstadt, Germany) supplemented with chloramphenicol (CHL) (OXOID, Basingstoke, United Kingdom) following the instructions of the manufacturer. Yeast cultures were incubated under aerobic conditions (ZHICHENG, Shanghai, China) at 37 °C for 24 h. The purity of the *H. pylori* cultures was checked by Gram-staining and urease, catalase and oxidase tests. The purity of the *Candida* strains was checked by Gram staining, reseeding in CHROMagar (Difco, Wokingham, UK) and the urease test.

### 2.3. Growth Curves of H. pylori and Candida Strains at 4 °C, 25 °C, 37 °C or 40 °C

In order to make sure to start the co-cultures of *H. pylori* and *Candida* strains when both microorganisms were in their respective exponential phase of growth, this assay was necessary to determine the incubation time required by each microorganism to reach the desired phase of growth. Each strain of either *H. pylori* or *Candida* spp. used in the present work was suspended at an optical density (OD) of 0.1 at 600 nm in Brucella broth (BB) (Difco, Wokingham, UK) supplemented with 5% FBS (BB-5%FBS), and 200 µL of each suspension were placed in a well of 96-well plates (Thomas Scientific, Swedesboro, NJ, USA) and plates incubated at 4 °C, 25 °C, 37 °C or 40 °C in an Infinite M200 PRO equipment (TECAN, Männedorf, Switzerland), in which the microaerobic conditions were obtained using CampyGen envelopes (Thermo Scientific, Waltham, MA, USA). The growth of each strain was monitored by OD at 600 nm every 8 h during 72 h for the bacterial strains and every 2 h during 50 h in the case of yeast strains. Additionally, in the case of *H. pylori,* 5 µL aliquots were obtained at each time OD was measured, and a Gram-staining was prepared to monitor possible morphological changes in bacterial cells caused by the incubation conditions.

### 2.4. Co-Cultures of H. pylori Strains with C. albicans Strains

Each *H. pylori* strain was co-cultured with each *Candida* spp. strain. A suspension of each strain of each kind of microorganism was independently prepared in 0.89% saline solution (SS) and adjusted to an OD of 0.1 at 600 nm. Then, 500 µL of each suspension of the bacterial strains were placed in wells of 12-well plates (Thomas Scientific, Swedesboro, NJ, USA) previously loaded with 4 mL de BB-5%FBS. Then, 500 µL of yeast cells from each strain were independently added to wells previously inoculated with *H. pylori*. Four wells were required for each bacterial strain in order to be independently co-cultured with each of the yeast strains. Plates containing the co-cultures were incubated under microaerobic conditions for 48 h at the following temperatures: 4 °C, 20 °C, 37 °C or 40 °C. Microaerobiosis atmosphere was achieved using CampyGen envelopes (Thermo Scientific, Waltham, MA, USA). All assays were repeated thrice. As controls for this assay, pure inocula of all *H. pylori* and *Candida* strains were used.

### 2.5. Search for Yeast Cells Containing Bacteria-Like Bodies (Y-BLBs)

During the 48 h incubation of the co-cultures, a 20 µL aliquot of each of them was obtained at times 0, 1, 3, 6, 12, 24 and 48 h and placed on a glass slide to prepare wet mounts for observation under an optical microscope, using the oil immersion 100× objective lens, (Leica, Wetzlar, Germany fitted with camera). For each co-culture, 200 yeast cells were counted and the percentage of yeast cells containing mobile bacteria-like bodies (Y-BLBs) was calculated. Additionally, to be used in the following assays, a sample from the co-cultures was added to 1 mL BB-5%FBS, vortexed (DLAB, Ontario, CA, USA) and 0.015 µg/mL clarithromycin (Sigma-Aldrich, St. Louis, MO, USA) added. This mixture was incubated under microaerobic conditions at 37 °C for 24 h to eliminate extracellular remaining *H. pylori* cells. Then, 20 µL of the culture were seeded on the surface of dishes containing Sabouraud agar (Merck, Darmstadt, Germany) plus 50 mg/L chloramphenicol (OXOID, Basingstoke, UK) (SA-CHL) and incubated for further 24 h at 37 °C under microaerobic conditions and colonies reseeded 6 times in SA-CHL to eliminate any residual extracellular *H. pylori* cells.

### 2.6. Identification of Intra-Yeast BLBs as H. pylori Using the FISH Technique

This procedure was completed according to Sánchez-Alonzo and coworkers [48] with modifications. Yeast cells previously co-cultured with *H. pylori* and showing BLBs were incubated at 37 °C for 24 h in SA-CHL to obtain yeast colonies free of extracellular bacteria. Cells from randomly selected colonies were transferred to tubes containing 1 mL 1X sterile phosphate-buffered saline (PBS) (Sigma-Aldrich, St. Louis, MO, USA) until reaching turbidity similar to tube 3 of the McFarland scale. Tubes were centrifuged at 6700× *g* for 2 min (Eppendorf, San Diego, CA, USA), washed once under the same conditions, the supernatant was discarded and 1 mL 1X PBS added to the pellet. The pellet was resuspended using a vortex (DLAB, Ontario, CA, USA) at medium speed for 10 s and 100 µL aliquots of each suspension placed on a glass slide. Smears were dried at room temperature for approximately 20 min and fixed using 200 µL formaldehyde solution (37%) (Sigma-Aldrich, St. Louis, MO, USA) and allowed to rest for 3 h at 4 °C in a humid chamber. Fixative was discarded and smears dehydrated with 1 mL of 50%, 80% and 96% ethanol (3 min each) and allowed to dry at room temperature. Then, 100 µL hybridization solution (270 µL NaCl 5M, 30 µL TRIS-HCl 1 M; 525 µL deionized formamide 37.7%, 675 µL MiliQ water and 1.5 µL SDS 10%) and 6 µL Hpy probe 5′-CACACCTGACTGACTATCCCG-3′ labeled with Cy3 [50] at a 5 ng µL^−1^ concentration were added to each smear. Slides were incubated in a thermoregulated bath (Elma, Singen, Germany) at 46–48 °C for 90 min in darkness and then 1 mL of preheated (46–48 °C) washing buffer (700 µL NaCl 5M, 1 mL 1M TRIS-HCl, 48.25 mL sterile distilled water and 50 µL SDS 10%) were added and incubated at the same temperature for further 20 min. After this time span, washing was repeated under the same conditions but incubated for only 5 min, and slides were allowed to dry at room temperature in the darkness. Once dried, 100 µL ConA-FITC 1 mg mL^−1^ (Sigma-Aldrich, St. Louis, MO, USA) was added to each smear and incubated for 10 min at room temperature in darkness, washed twice using 1 mL 1X PBS and allowed to dry in the darkness. Smears were observed using a fluorescence microscope fitted with a camera and filters FITC (AT480/535) and TRIC (AT540/605) (Motic, Viking Way, Richmond, VA, Canada). Images were processed and merged using the ImageJ software (NIH Image, Bethesda, MD, USA). For this assay, pure cultures of *H. pylori* H707 and *C. glabrata* LEO-37 were used as positive control and negative control, respectively.

### 2.7. Detection of the H. pylori 16S rRNA Gene in Total DNA Extracted from Y-BLBs

Y-BLBs were obtained as described above (FISH assay). Total DNA of yeast cells was obtained by means of the UltraClean Microbial DNA Isolation kit (MO BIO, Carlsbad, CA, USA), following the instructions of the manufacturer. To amplify the *H. pylori 16S rRNA* gene, 0.2 mL Eppendorf tubes (Eppendorf, Framingham, MA, USA) were loaded with 12.5 µL master mix (TAKARA BIO INC, Shiga, Japan), 1 μL specific primers F-5′CTCGAGAGACTAAGCCCTCC3′ and R-5′ATCTGACGCTGATGTGC3′ [11], 1.5 μL yeast DNA and 5.5 µL PCR grade water to obtain a final volume of 25 µL. An Eppendorf thermocycler (Eppendorf, Framingham, MA, USA) was programmed for 30 PCR amplification cycles using an initial denaturation temperature of 94 °C for 1 min, denaturation temperature of 98 °C for 30 s, hybridization temperature of 53 °C for 5 s and extension temperature of 72 °C for 40 s and a final extension temperature of 72 °C for 10 min. Then, electrophoresis was performed in a 2% agarose gel (Lonza, Walkersville, MD, USA) plus 1 µL GelRed (Biotium, Fremont, CA, USA) at 70 V for 90 min. Amplicons were observed under UV light using an ENDURO photo documentation system (Labnet, Edison, NJ, USA). As control for this assay, DNA from *H. pylori* SS-1 was used as positive control and DNA from *C. albicans* ATCC 90028 as negative control.

### 2.8. Cell Viability Assay

In 24 h cultures in SA of yeasts which were positive for the presence of BLBs after being co-cultured for 48 h with *H. pylori*, yeast colonies were randomly selected, suspended in 1 mL of SS and adjusted to turbidity similar to 0.5 of the McFarland scale. Then, 1 µL working solution of the LIVE/DEAD BacLight Bacterial Viability Kit L-7012 (ThermoFisher, USA) was added, and the suspension was incubated for 15 min in the darkness. Then, suspensions were vortexed (DLAB, Ontario, CA, USA) at minimal speed for 3 s. Posteriorly, 10 µL of the suspension was placed on a glass slide and observed using the 100× objective lens of a fluorescence microscope fitted with a camera (Motic, Viking Way, Richmond, VA, Canada). The filters used were FITC (AT480/535) and TRIC (AT540/605). Images were processed using the ImageJ (NIH Image, USA) software for merging them.

### 2.9. Statistical Analysis

SPSS 24.0 software (IBM Company, Armonk, NY, USA) was used to process the data obtained. Tukey’s test was applied to determine the presence of statistically significant differences. Values of *p* ≤ 0.05 were considered significant, and values of *p* ≤ 0.0001 were considered highly significant. According to the results of Tukey´s test, data shown in tables or figures sharing the same letter are not significantly different.

## 3. Results

### 3.1. Growth Curves of H. pylori and Candida Strains at Different Temperatures

**Figure 1:**
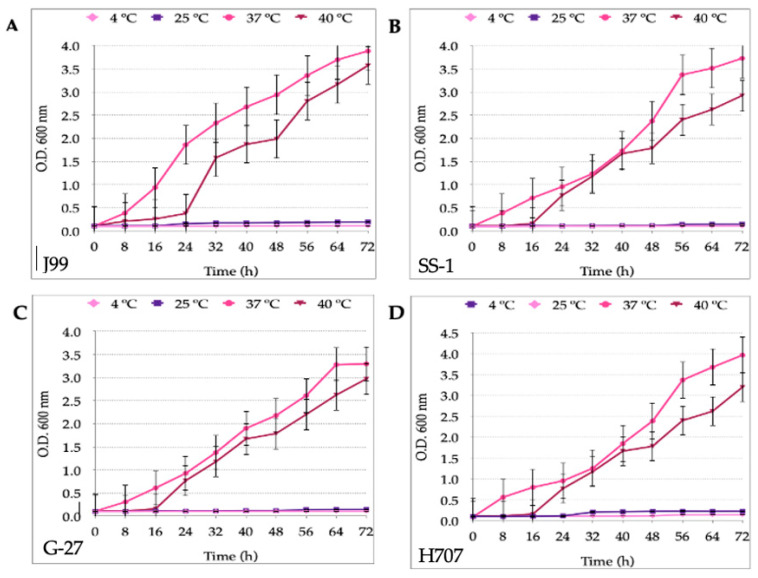
Growth curves of *H. pylori* strains (**A**) J99, (**B**) SS-1, (**C**) G-27 and (**D**) H707 incubated at 4 °C, 25 °C, 37 °C or 40 °C in Brucella broth supplemented with 5% bovine serum. Results are expressed as mean ± SD. OD: optical density.

In order to start the *H. pylori*-*Candida* co-cultures with both microorganisms in their exponential phase, it was necessary, as a first step, to determine the growth curves of the four *H. pylori* and of the four *Candida* strains. The growth curves of the *H. pylori* strains (J99, SS-1, G-27 and H707) cultured at 4 °C, 25 °C, 37 °C or 40 °C showed that the strains only grew when incubated at 37 °C or 40 °C (Figure 1). There were significant differences when the growth at the two higher temperatures was compared to that of the two lower temperatures. All four strains showed no significant difference when incubated at 37 °C or 40 °C (*p* = 0.07). Nevertheless, the exponential phase started earlier when incubated at 37 °C (8 h) than at 40 °C (16 h). On the other hand, Gram staining showed a larger number of coccoid *H. pylori* cells when incubated at 40 °C compared to the incubation at 37 °C (Figure S1).

**Figure 2:**
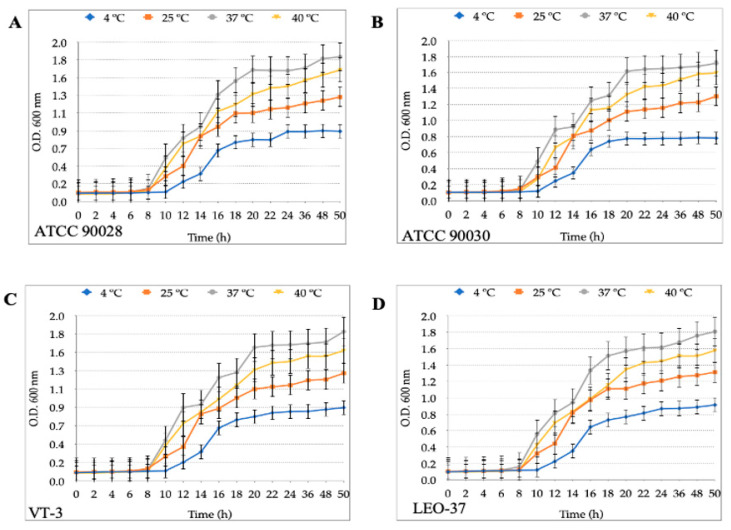
Growth curves of the four strains of *Candida* assayed: (**A**) *C. albicans* ATCC 90028 (**B**) *C. glabrata* ATCC 90030, (**C**) *C. albicans* of vaginal origin VT-3 and (**D**) *C. glabrata* of oral origin LEO-37 cultured at 4 °C, 25 °C, 37 °C or 40 °C in Brucella broth supplemented with 5% fetal bovine serum. Results are expressed as mean ± SD. OD: optical density.

Regarding the growth curves of the *Candida* strains, all four of them (*C. albicans* ATCC 90028, *C. glabrata* ATCC 90030, *C. albicans* of vaginal origin VT-3 and *C. glabrata* of oral origin LEO-37) grew when incubated at 25 °C, 37 °C or 40 °C, showing no significant differences at these three temperatures. When incubated at 4 °C, all four *Candida* strains showed a short exponential growth phase and an extended stationary phase starting at 18 h of incubation (Figure 2). No significant differences were observed when comparing the growth of each yeast strain at 37 °C and 40 °C (*p* = 1.9) or at 40 °C and 25 °C (*p* = 1.0). When comparing the growth curves obtained at 25 °C with those obtained at 37 °C, no significant difference was observed. A significant decrease was observed when the growth at 4 °C was compared to that at 25 °C, 37 °C or 40 °C (*p* = 0.009).

### 3.2. Identification of Bacteria-Like Bodies (BLBs) within Yeast Cells after Co-Culturing Both Microorganisms at Different Temperatures

**Figure 3:**
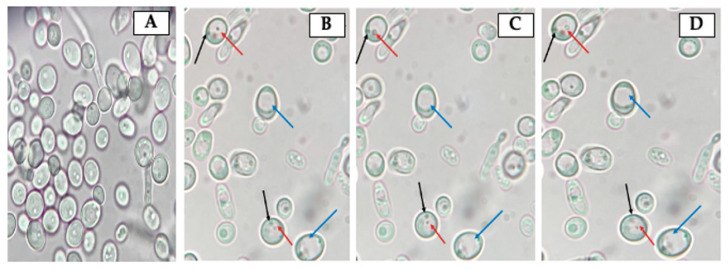
Micrographs, obtained at 1 s intervals, of wet mounts from (**A**) pure culture of *C. albicans* VT-3 (yeast purity control), (**B**–**D**) *H. pylori* G-27 and *C. albicans* VT-3 co-cultures incubated at 37 °C, showing the change in position of bacteria-like bodies (BLBs) (red arrows). Additionally, it is possible to observe yeast cells lacking BLBs (blue arrows) and nuclei of yeast cells (black arrows). The movement of BLBs can be observed in Video S1.

**Figure 4:**
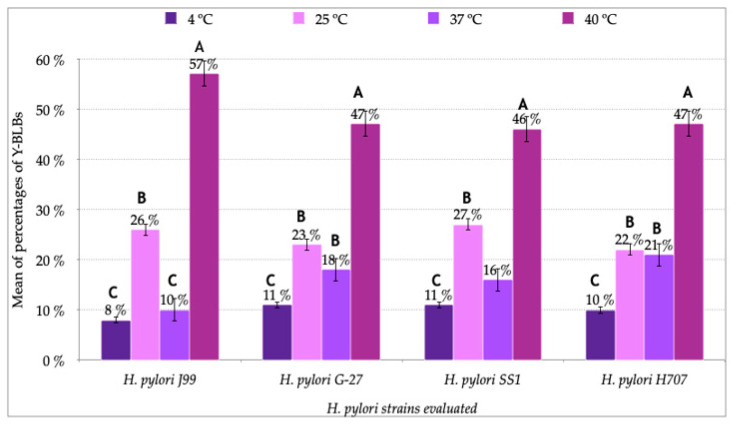
Average of the mean percentages of yeasts cells harboring bacteria-like bodies (Y-BLBs) of the four *Candida* strains after co-incubation of *H. pylori* strains with *Candida* strains at 4 °C, 25 °C, 37 °C or 40 °C during 48 h. The highest percentage of BLBs was found in co-cultures incubated at 40 °C. It can be seen that the highest percentages were obtained with the co-cultures carried out with the *H. pylori* J99 strain in microaerobic conditions. Results are expressed as mean ± SD. Means with different letters are significantly different (*p* < 0.05).

The observation of fresh mounts of aliquots obtained from *H. pylori* and *Candida* strains co-cultures incubated at 4 °C, 25 °C, 37 °C or 40 °C for 48 h allowed detecting the presence of Y-BLBs (Figure 3). The mean of Y-BLBs percentages obtained when co-incubating *H. pylori* strains with *Candida* strains was calculated considering the global number of Y-BLBs per incubation temperature and the *H. pylori* strain being evaluated. The quantification of Y-BLBs indicated that the larger Y-BLBs percentage was present in co-cultures incubated at 40 °C (46 to 57%) followed by those incubated at 25 °C (22 to 27%), 37 °C (10 to 21%) and finally those at 4 °C (8 to 11%) (Figure 4). It was also observed that co-cultures combining the *H*. *pylori* J99 strain with all *Candida* strains incubated at 25 °C and 40 °C showed the largest percentages of Y-BLBs, but not at the rest of the temperatures assayed (Figure 4). The wet mounts of co-cultures also showed *H. pylori* cells adhered to hyphae of yeasts (a Gram staining demonstrating this observation is shown in Figure S2).

**Figure 5:**
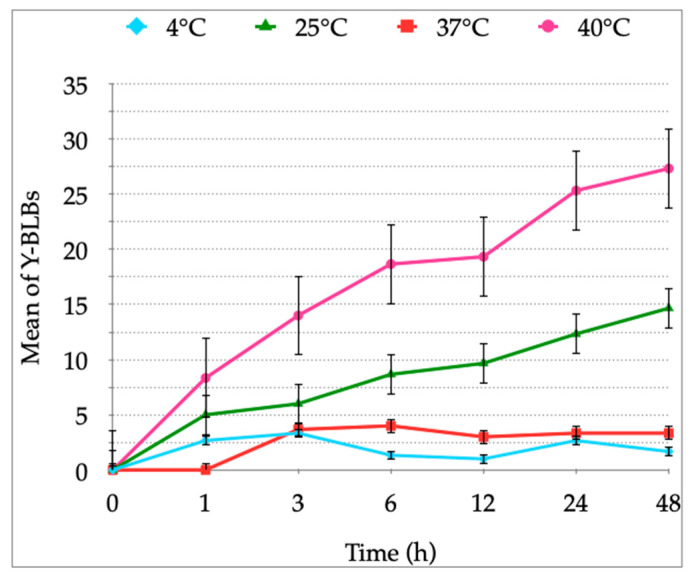
Means of yeasts harboring bacteria-like bodies (Y-BLBs) obtained in *H. pylori* J99 and *C. albicans* ATCC 90028 co-cultures incubated for up to 48 h at 4 °C, 25 °C, 37 °C or 40 °C in Brucella broth supplemented with 5% fetal bovine serum. After calculating the Y-BLBs means for all combinations of *H. pylori* and *Candida* co-cultures, similar patterns were observed. Results are expressed as mean ± SD.

In the co-cultures incubated at 4 °C, 25 °C or 40 °C, Y-BLBs were observed from the first hour of co-culture, but in the co-cultures incubated at 37 °C, Y-BLBs were observed after 3 h of co-culture. The averages of the mean Y-BLBs calculated after incubating the co-cultures at 4 °C or 25 °C were lower than those obtained when incubating at 37 °C or 40 °C (Figure 5). Figure 5 also shows that the higher means of Y-BLBs when co-incubating *H. pylori* J99 and *C. albicans* ATCC 90028 were achieved when co-cultures were incubated at 40 °C for 48 h. This same pattern was similar for all the possible combinations of co-cultures (data not shown). This assay was completed in triplicate.

**Figure 6:**
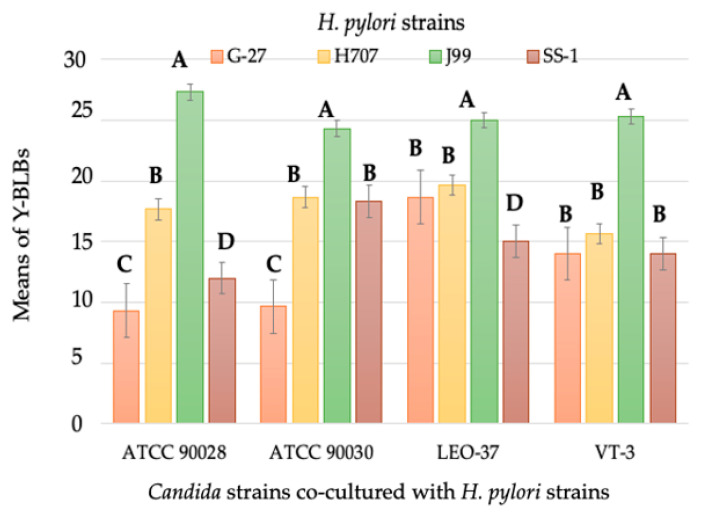
Means of yeasts harboring bacteria-like bodies (Y-BLBs) in *H. pylori* and *Candida* co-cultures incubated at 40 °C for 48 h. The higher means of Y-BLBs were observed when the *H. pylori* J99 strain was co-incubated with any of the four yeast strains assayed. Results are expressed as mean ± SD. Means with different letters are significantly different (*p* < 0.05).

After determining that the differences in percentages of Y-BLBs depended on the bacterial strain and temperature (Figure 4), data were analyzed to ascertain if the endosymbiotic relationship also depended on the yeast strain used in the co-cultures. Based on the information provided in Figure 5, it was concluded that the larger percentages of Y-BLBs were present at 48 h of co-culturing at 40 °C. Data obtained in that particular incubation time were considered in order to analyze in which bacterium-yeast combination the higher Y-BLBs were obtained. The co-cultures, including the *H. pylori* J99 strain, showed the higher Y-BLBs means when co-incubated with any of the yeast strains assayed in the present work. (Figure 6). After analyzing if the symbiotic relationship also depended on the yeast strain, no significant difference was found to sustain this possibility (Table S1).

### 3.3. Confirmation, by FISH, That BLBs Correspond to Intra-Yeast H. pylori

**Figure 7:**
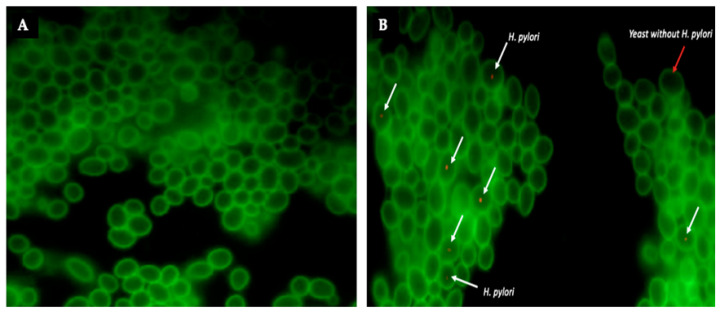
FISH identification of intra-yeast *H. pylori.* (**A**) Pure culture of *C. glabrata* ATCC 90030, (**B**) *H. pylori* J99-*C. glabrata* ATCC 90030 co-culture after hybridization to detect *H. pylori* using the Hpy 5′-CACACCTGACTGACTATCCCG-3′ probe labeled with Cy3 (white arrows). Red arrow indicates a yeast cell lacking bacteria-like bodies. Green fluorescence corresponds to the ConA-FITC fluorochrome bind to chitin of the yeast cell wall.

FISH results confirmed that BLBs moving within the vacuoles of yeast cells revealed by the wet mounts are *H. pylori* cells. This conclusion is based on the red fluorescence emission emitted by the *H. pylori*-specific fluorescent probe that hybridized with the bacterial DNA within the yeast (Figure 7). Besides intra-yeast *H. pylori* detected by FISH, no other corpuscles were detected within yeast cells.

### 3.4. Amplification of the H. pylori 16S rRNA Gene from Total DNA Extracted from Yeast Cells Previously Co-Cultured with the Bacterium

**Figure 8:**
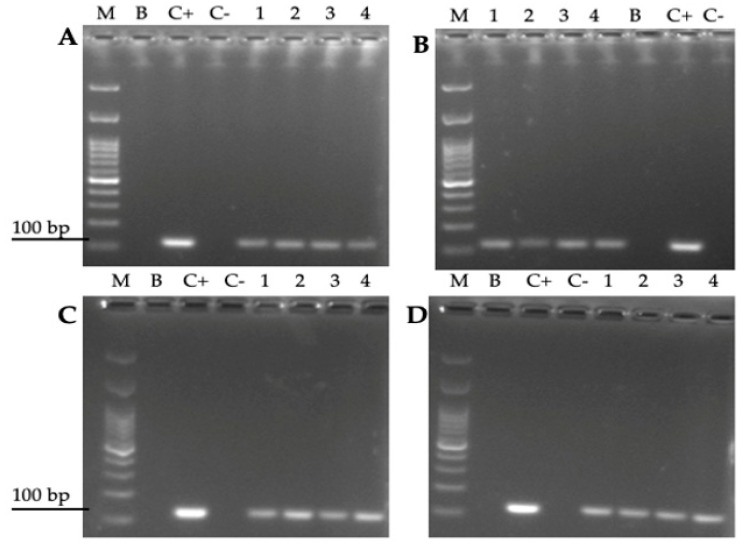
Agarose gel (2%) showing amplicons obtained after PCR amplification of the *H. pylori 16S rRNA* gene from total DNA extracted from yeast cells after co-incubating bacterial and yeast cells for 24 h at (**A**) 4 °C, (**B**) 25 °C, (**C**) 37 °C or (**D**) 40 °C. M: molecular weight marker, B: blank (master mix, primers, PCR grade water), C-: negative control (pure *C. albicans* ATCC 90028 DNA), C+: positive control (pure *H. pylori* SS-1 DNA), lane 1: *H. pylori* J99-*C. albicans* ATCC 90028 co-culture, lane 2: *H. pylori* J99-*C. glabrata* ATCC 90030 co-culture, lane 3: *H. pylori* J99-*C. albicans* VT-3, lane 4: *H. pylori* J99-*C. glabrata* LEO-37.

To support the results obtained by FISH, an assay was performed to amplify by PCR the rRNA *16S* gene of *H. pylori* from total yeast DNA in which mobile BLBs were observed. All samples of yeast DNA in which the presence of BLBs was previously observed showed, in agarose gels, the presence of the amplicon of the expected size for the above-mentioned *H. pylori* gen (Figure 8). The same amplicon was also present in DNA extracted from pure *H. pylori* and absent in the DNA extracted from pure yeast (Figure 8). The presence of the *H. pylori 16S rRNA* gene was detected in all yeasts isolated from co-cultures in which Y-BLBs were observed, and *H. pylori* was identified by the FISH technique.

### 3.5. Cell Viability Assays

**Figure 9:**
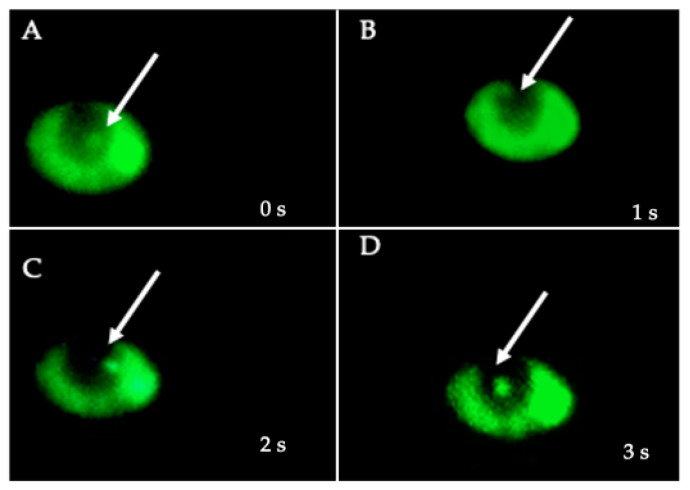
Viability assay micrographs, obtained at 1 s intervals ((**A**) time 0 s, (**B**) time 1 s, (**C**) time 2 s, (**D**) time 3 s) a viable (green fluorescence) *H. pylori* J99 cell within the vacuole of *C. glabrata* LEO-37 strain yeast cell. Images depict the change in the position of the *H. pylori* cell within the vacuole of the yeast cell caused by the movement of the bacterium.

After confirmation that BLBs corresponded to intra-yeast *H. pylori* using FISH and PCR techniques, the viability of yeast and bacterial cells was evaluated using the LIVE/DEAD BacLight Bacterial Viability kit in order to confirm that the movements of BLBs previously observed in the wet mounts prepared from the co-cultures corresponded to viable *H. pylori* cells within viable yeast cells. The green fluorescence present inside the vacuole of yeasts, caused by the SYTO 9 of the kit, demonstrates the viability of the intra-yeast bacteria (Figure 9).

## 4. Discussion

Temperature is an abiotic factor of importance for the diversity and the metabolism of organisms, including bacteria and fungi. Temperature directly affects microbial growth as well as fructification, sporulation, spore germination, motility and survival of fungi [51,52]. Therefore, each microorganism needs to remain within its appropriate range of temperature to survive. In fact, certain microorganisms can colonize environments whose temperature is below 0 °C, while others colonize niches whose temperature is close to 100 °C [52]. During their life, microorganisms can be subjected to temperature fluctuations, changes to which they must rapidly adapt in order to survive.

*H. pylori* is considered a mesophilic bacterium whose growing temperature range is 35–37 °C, and outside this range, it acquires a coccoid morphology corresponding to its VBNC condition [26,53]. This optimal temperature range is apparent when observing the growth curves reported in the present work for the four *H. pylori* strains assayed, which grew better at 37 °C than at 4 °C, 25 °C or 40 °C. When incubated at 40 °C, the growth of the four *H. pylori* strains assayed was not significantly different to that shown at 37 °C, results supported by the literature indicating that its growth at 42 °C tends to be variable [53]. Therefore, although it was incubated at a temperature above its optimal range, it was not surprising that this bacterium grew at 40 °C. It has been reported that when it finds itself outside its optimal temperature range, *H. pylori* will adopt its VBNC condition (coccoid morphology) [54]. This information, combined with our results, suggests that *H. pylori* is capable of surviving at 40 °C, but the exposure to this temperature is a stressing factor that induces the morphology change to the coccoid shape. This explains why our Gram-staining observations showed more *H. pylori* coccoid cells when incubation was completed at 40 °C as compared to incubation at 37 °C. On the contrary, no growth was observed when *H. pylori* was incubated at 25 °C or 4 °C. The statistical analysis of the optical densities of these two lower temperatures showed no significant in bacterial growth (measured assessing OD). These results are in accordance with those reported in the literature indicating that *H. pylori* may remain viable at these temperatures but does not replicate [4,53,55].

Regarding yeasts of the *Candida* genus, they grow within a wider range of temperatures than *H. pylori* (20–42 °C) [56]. Therefore, it was expected that the yeast strains assayed in this work grew at 25 °C, 37 °C or 40 °C. On the other hand, the growth of yeast cells was significantly reduced when incubated at 4 °C, a reasonable consequence of the slowing down of their metabolism, which affects their growth rate. Since yeast cells subjected to low temperatures (10 °C or less) activate an adaptative response which allows them to survive at temperatures close to freezing [57], it is possible to explain the growth, although significantly reduced, of *Candida* cells at 4 °C reported in this work.

The presence of BLBs within yeast cells previously co-incubated with *H. pylori* cells was observed in all the co-cultures combining pairs of each one of the four bacterial strains with each one of the four yeast strains. Numbers of Y-BLBs obtained in the cultures incubated at 37 °C, optimal for the growth of *H. pylori* cells and yeast cells belonging to the genus *Candida* [53,56], were used as a pattern to be compared with numbers of Y-BLBs obtained at the other temperatures assayed. The percentages of Y-BLBs at 37 °C was 10–21%, indicating that *H. pylori* harbors into a low percentage of yeast cells in the absence of an apparent stressing factor. This observation is in agreement with the results of another report of our laboratory in which we analyzed if pH variations, as a stressing factor, affected the harboring of *H. pylori* cells within yeast cells. The results of that report allowed concluding that although *H. pylori* cells enter into yeast cells under optimal culture conditions, the percentages of Y-BLBs increased significantly at stressing acidic pH values (pH 3, pH 4) [49]. When co-cultures were incubated at temperatures non-optimal for *H. pylori*, the percentages of Y-BLBs increased when co-cultures were incubated at 40 °C (46–57%) or 25 °C (22–27%), but it was significantly reduced when subjected to 4 °C (8–11%). The presence of Y-BLBs in co-cultures incubated at temperatures of 25 °C or 4 °C and the lack of growth of *H. pylori* here reported at those temperatures confirms previous reports indicating that those are stressing temperatures for this bacterium [5,53]. Thus, it is possible to suggest that *H. pylori* harbors within yeast cells before its viability or its morphology are affected by a stressing temperature. There was a significant difference in the means of Y-BLBs when comparing co-cultures incubated at 4 °C or 25 °C (*p* = 0.002), being that the Y-BLBs were fewer at 4 °C. A possible explanation for this difference could be that *H. pylori* loses motility and enters into the coccoid morphology at 4 °C faster than at temperatures close to 25 °C, permitting more Y-BLBs to occur at the higher of these two temperatures [58].

It has been reported that temperature also affects the morphogenesis of *C. albicans*, influencing its change from blastoconidia to pseudohypha or hypha, filamentous morphologies having adhesins that favor the adhesion of the yeast to cells of the host [59,60]. Although so far there are no reports describing hyphal receptors or adhesins involved in the internalization of *H. pylori,* we observed a large number of *H. pylori* cells adhered preferentially to hyphae of *C. albicans*. There are reports indicating that hyphae are structures rich in nutrients and in lipidic membranes plentiful in ergosterol, a sterol analogous to cholesterol, a lipid for which *H. pylori* has high affinity [59,60], suggesting that this bacterium may have an affinity for ergosterol to use it in its metabolism. Nevertheless, more evidence is required to sustain this hypothesis. Finally, the behavior of the strains of each microorganism in the co-cultures was statistically analyzed, and it was concluded that the *H. pylori* and yeast cells endosymbiotic relationship was dependent on the bacterial strain and on the temperature inducing the harboring of *H. pylori* cells within yeast cells. When co-incubated with all yeast strains, the strain *H. pylori* J99 produced the largest percentages of Y-BLBs, independent of the yeast strain with which it was co-incubated. Although this strain showed larger percentages of Y-BLBs when co-incubated with *C. albicans* ATCC 90028, no significant differences were found when compared to the rest of the yeast strains. No significant differences were observed when the means of Y-BLBs resulting from co-culturing *H. pylori* J99 and *C. albicans* ATCC 90028 strains were compared with the Y-BLBs means resulting from the co-cultures of the *H. pylori* J99 strain with the other *Candida* strains.

On the other hand, co-cultures in which *C. glabrata* strains were used also showed high percentages of Y-BLBs; nevertheless, yeasts belonging to this fungal species do not form hyphae [61]. Therefore, the harboring of *H. pylori* cells within yeasts of this species may be facilitated by other characteristics of the yeast cells of this species. It has been described that *C. glabrata* possesses approximately 67 types of adhesins, components of their cell surface, which, among their functions, include biofilm formation, including mixed bacteria–yeast biofilms [62]. It has also been reported that yeasts belonging to the *Candida* genus co-aggregate with a number of bacteria, including *Staphylococcus aureus*, *Enterococcus faecalis*, *Prevotella intermedia* and *Porphyromonas gingivalis* [63]. Moreover, Ansorg and coworkers [44] reported the adherence of *H. pylori* to the surface of *Candida* spp. cells, particularly those not belonging to the species *C. albicans*, when not morphologically blastoconidia. Thus, to bring light to the endosymbiotic relationship between *H. pylori* and cells of yeasts belonging to the genus *Candida,* further in-depth research is required.

## 5. Conclusions

*H. pylori* showed a significant growth at 40 °C, a temperature at which it also increased its harboring within yeast cells belonging to the *Candida* genus. Under the conditions of culturing used in this study, the endosymbiotic relationship between these two microorganisms is dependent on the *H. pylori* strain. It can also be concluded that *H. pylori* retains its viability when harbored within *Candida* spp. cells. Temperatures below the range considered as optimal for *H. pylori*, such as 4 °C or 25 °C, generate evident stress for this bacterium, but they did not favor the entry of bacteria into yeast cells because no significant percentages of Y-BLBs were detected at these temperatures. Therefore, *H. pylori* is less efficient at harboring within yeast cells at low temperatures, which rapidly affects its morphology or viability more than at high ones.

## Data Availability

Not applicable.

## Supplementary Materials

The following are available online at https://www.mdpi.com/article/10.3390/biology10090915/s1, Figure S1: Gram staining of *H. pylori* J99 cultures incubated at 37 °C (A) or 40 °C (B) showing the increased number of coccoid cells when incubated at 40 °C.; Gram-staining of *H. pylori* H707-*C. albicans* VT-3 co-culture showing the co-aggregation of both microorganisms. Observe the preference of *H. pylori* cells for filamentous structures (pseudohyphae or hyphae). Figure S2: Gram-staining of *H. pylori* H707-C. albicans VT-3 co-culture showing the co-aggregation of both microorganisms. Observe the preference of *H. pylori* cells for filamentous structures (pseudohyphae and hyphae). Table S1: Analysis to evaluate if there were significant differences in Y-BLBs means when strains of *H. pylori* were incubated with *Candida* strains in 48 h co-cultures incubated at 40 °C, Video S1: Movement of intra-yeast bacteria-like bodies (BLBs) observed in a wet mounting. (https://youtu.be/Mj-KGUsdqUg, accessed on 10 August 2020)
