# Supplementary material for: Temperatures Outside the Optimal Range for Helicobacter pylori Increase Its Harboring within Candida Yeast Cells
Source: Biology (Basel). 2021 Sep 15;10(9):915. doi: 10.3390/biology10090915 (PMC8472035; doi:10.3390/biology10090915)
Supplement: Supplementary file 1 [file biology-10-00915-s001.zip › biology-1335788-supplementary.pdf]

biology

MDPI

Supplementary figures and table:

Table S1: Analysis to evaluate if there were significant differences in Y-BLBs means when strains of *H. pylori* were incubated with *Candida* strains in 48 h co-cultures incubated at 40 °C.

	Candida strains						
H. pylori strains	ATCC 90028	ATCC 90030	LEO-37	VT-3	Mean	Tukey's test	
G-27	9.6667	9.6667	18.3333	11.6667	**12.3333**	ATCC 90028	**A**
H707	15.3333	14.6667	20.3333	15.6667	**16.5000**	ATCC 90030	**A**
J99	25.3333	23.0000	24.6667	24.6667	**24.4167**	LEO-37	**A**
SS-1	12.3333	15.6667	15.3333	13.6667	**14.2500**	VT-3	**A**
**Mean**	**15.6667**	**15.7500**	**19.6667**	**16.4167**			

ANOVA: Single Factor

Means sharing the same letter were not significantly different ($p > 0.05$) according to the Tukey test.

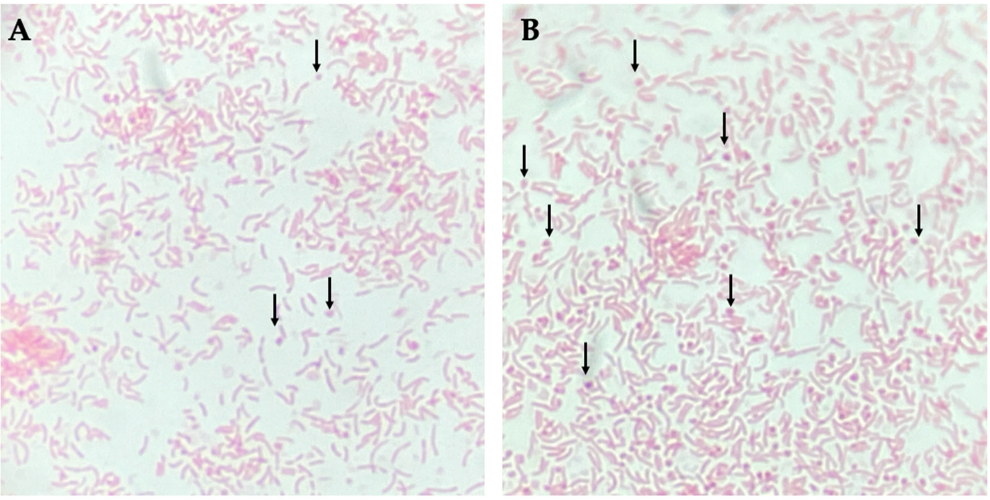

Figure S1: Gram staining of *H. pylori* J99 cultures incubated at A) 37 °C or B) 40 °C showing the increased number of coccoid cells when incubated at 40 °C.

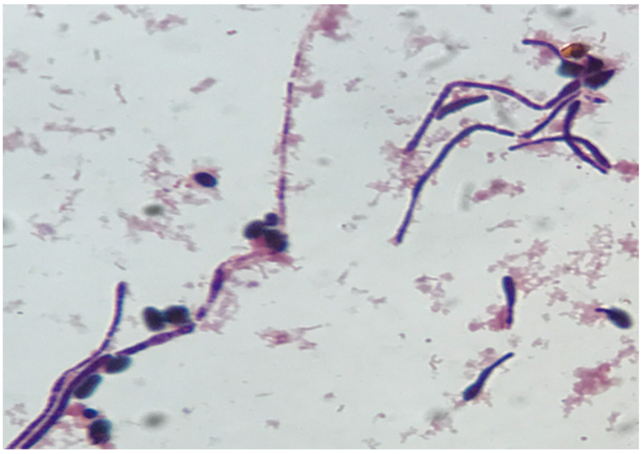

Figure S2: Gram-staining of *H. pylori* H707-*C. albicans* VT-3 co-culture showing the co-aggregation of both microorganisms. Observe the preference of *H. pylori* cells for filamentous structures (pseudohyphae and hyphae).
